# GePMI: A statistical model for personal intestinal microbiome identification

**DOI:** 10.1038/s41522-018-0065-2

**Published:** 2018-09-04

**Authors:** Zicheng Wang, Huazhe Lou, Ying Wang, Ron Shamir, Rui Jiang, Ting Chen

**Affiliations:** 1 MOE Key Laboratory of Bioinformatics and Bioinformatics Division, BNLIST and Department of Automation, Tsinghua University, 100084 Beijing, China; 2 Bioinformatics Division, BNLIST and Department of Computer Science and Technology, Tsinghua University, 100084 Beijing, China; 3 Department of Automation, Xiamen University, 361005 Fujian, China; 4 Blavatnik School of Computer Science, Tel-Aviv University, Tel Aviv, Israel

## Abstract

Human gut microbiomes consist of a large number of microbial genomes, which vary by diet and health conditions and from individual to individual. In the present work, we asked whether such variation or similarity could be measured and, if so, whether the results could be used for personal microbiome identification (PMI). To address this question, we herein propose a method to estimate the significance of similarity among human gut metagenomic samples based on reference-free, long *k*-mer features. Using these features, we find that pairwise similarities between the metagenomes of any two individuals obey a beta distribution and that a *p* value derived accordingly well characterizes whether two samples are from the same individual or not. We develop a computational framework called GePMI (Generating inter-individual similarity distribution for Personal Microbiome Identification) and apply it to several human gut metagenomic datasets (>300 individuals and >600 samples in total). From the results of GePMI, most of the human gut microbiomes can be identified (auROC = 0.9470, auPRC = 0.8702). Even after antibiotic treatment or fecal microbiota transplantation, the individual *k*-mer signature still maintains a certain specificity.

## Gut microbes: Identifying our personal microbiomes

A computational method for processing the results of genetic analysis of gut microbes can discriminate between and identify the microbes populating different individuals. The pool of microbial genomes in the gut — the gut microbiome — comes from many different microbes. Researchers and clinicians are keenly investigating the significance of these gut microbes in health and disease. Ting Chen and Rui Jiang at Tsinghua University in Beijing, with co-workers elsewhere in China and in Israel, developed a method they call ‘Generating inter-individual similarity distribution for Personal Microbiome Identification’ (GePMI) to classify microbiomes. The procedure can reliably identify different people’s microbiomes, even after antibiotic treatment or the administration of donor microbes in fecal transplantation. GePMI will help monitor the variation among microbiomes and hopefully reveal associations linking gut microbes with specific diseases.

## Introduction

Recent studies have shown that the human gut microbiome should be regarded as a second genome independent of, but interacting with, both the host human genome and the environment.^1-5^ Many diseases are associated with human gut microbiomes,^6^ including obesity,^7^ diabetes,^8,9^ inflammatory bowel disease,^10^ liver cirrhosis,^11^ cancers,^12-14^ and mental illness.^15^ The human microbiome shows vast genetic diversity, and in spite of reports that it shares many core microbes among most individuals,^16^ the concept that there is a core set of species in the microbiota is becoming more unlikely.^17^ Enterotypes, which classify living organisms based on their bacteriological ecosystem in the gut microbiome, were previously proposed to cluster microbiomes into a few groups.^18^ However, subsequent analysis demonstrated that enterotypes should not be considered as distinct clusters but rather as densely populated areas in the compositional landscape.^19^

On the other hand, an individual’s microbiome is dynamic and constantly changing^20-22,23^ owing to environmental variables, such as human health and diet.^24-26^ In general, however, a microbiome maintains long-term stability.^27,28^ Experimental results have shown that the taxonomic compositions of two metagenomic samples from the same person are not always the same. Moreover, as time between taking the two samples from an individual increases, the difference tends to increase.^20,29^ Nonetheless, the difference between the microbiomes of any two individuals is greater than that between two samples from the same individual.^30,31^ Therefore, we ask if it is possible to distinguish the microbiome of one unique individual from that of others. If so, this would indicate the presence of invariants in an individual’s microbiome despite its dynamic nature.

To uniquely identify individual microbiomes, Franzosa et al. proposed the concept of metagenomic codes.^30^ They constructed a personal unique code set by using a combination of operational taxonomic units (OTUs) and species-specific marker genes from microbial reference genomes. The code set then functions as a fingerprint to uniquely identify a person. They showed that using additional appropriate features in a particular population can have favorable results. However, this approach faces a major challenge: to extract sufficient sequence information for personal identification^32^ from the huge amount of metagenomic sequences in a large population.

In this paper, we propose a fast, accurate, and reference-free method called GePMI (generating inter-individual similarity distribution for personal microbiome identification) for individual microbiome identification. GePMI extracts only kilobytes of sequence information from gigabytes of metagenomic sequences and uses it to distinguish an individual’s microbiome from the others’ with high accuracy.

Our approach recognizes extensive variation in the abundance of each microbe in a microbiome. However, our hypothesis holds that genome sequences at strain level, specifically single-nucleotide polymorphisms,^33^ indels (insertions and deletions), and structural variants,^11,34,35^ remain highly host-specific and stable. We propose to extract long *k*-mers as features^36^ to capture such genetic diversity in metagenomes instead of following the time-consuming strategy of genome assembly and read mapping,^37^ essentially because long *k*-mers are mostly unique in metagenomes and contain more specific genetic information compared to short *k*-mers.^38^ Thus we represent each metagenomic sequencing sample with a *k*-mer set, the *k*-mers that are present in the sample, and use the MinHash technique^39^ to measure the Jaccard similarity between two metagenomic samples.^40-43^ We show that most metagenomic samples from the same individual are significantly similar to each other but not to those from different individuals, even after dramatic environmental perturbations, such as antibiotic treatment^44,45^ or fecal microbiota transplant (FMT).^46-48^

For each sample, GePMI computes an inter-individual similarity distribution and uses it to test whether a query sample and the given sample come from the same individual. We tested GePMI over a large set of metagenomic data consisting of 612 samples from 155 individuals with multiple sampling visits and 146 individuals with only one sample. We demonstrated that the precision of PMI is improved by 10% by using GePMI compared with directly using samples’ similarity, and if we set a proper significance threshold for GePMI, we can almost eliminate all false positives, even for some individuals who underwent medical treatments, including antibiotic treatment and FMT. Although these treatments significantly altered the microbial community, >85% of the samples could still be accurately identified by GePMI. These results showed that GePMI can characterize the personal microbiome with accuracy, reliability, and efficiency.

## Results

### Overview of GePMI

If two metagenomic samples are taken from the same individual at different times, we hypothesize that their similarity will be much higher, while samples collected from other individuals at different times will have lower similarity.^29^ In GePMI, we use the MinHash function to approximate the Jaccard similarity for pairwise similarity calculation, and we fit a beta distribution to determine the significance of the similarity in order to evaluate whether two samples originated from the same individual.

For each metagenomic sample, we down-sampled the dataset to eliminate the impact of different sequence depths and split reads into *k*-mers. In general, *K* ≥ 15 can be regarded as long,^49^ and here we tested several values of *k* to balance the auROC (area under receiver operating characteristic) and auPRC (area under precision-recall curve). In GePMI, we used sourmash^39^ for similarity calculation and showed the effects of different choices of *k* on the results.

**Fig. 1:**
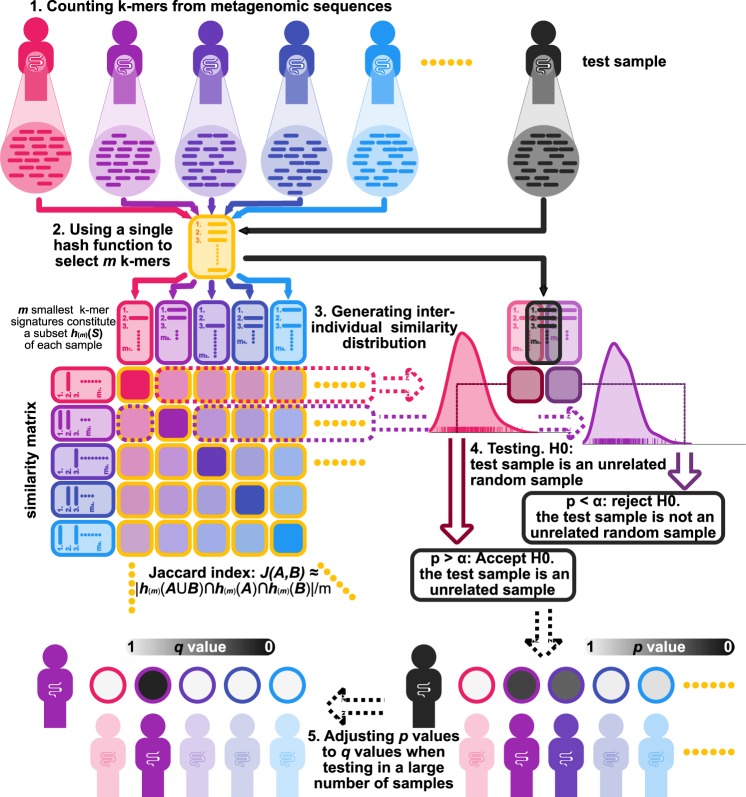
Overview of GePMI. (1) Each metagenomic sequencing dataset is processed into a *k*-mer set. (2) Each *k*-mer set is hashed into a subset of size m using the MinHash function so that the Jaccard similarity of the two *k*-mer sets can be approximated by MinHash similarity. (3) Each sample is then compared with other samples from unrelated individuals to generate a similarity distribution, which can be fitted by a beta distribution. (4) A query sample can be tested against each distribution. If its *p* value is below a threshold, it will be assigned to the sample with that distribution. (5) When testing in multiple distributions, *p* values are adjusted to control the false discovery rate

For each collected sample, we pre-computed its similarity scores with other samples from unrelated individuals to generate an inter-individual similarity distribution for this sample, which was fitted into a beta distribution. For a newly acquired metagenomic sample, we tested it against the fitted distribution of a collected sample with the null hypothesis that the two samples are from unrelated individuals. If the *p* value is small enough, we reject this hypothesis, accepting that the test sample belongs to the same individual. When a sample is queried against many collected individuals, we control the multiple testing using the false discovery rate (FDR) (Fig. 1).

### Performance of GePMI

We collected 612 metagenomic samples covering 301 individuals from five datasets, namely, Human Microbiome Project (HMP), Metagenomics of the Human Intestinal Tract (MetaHIT), microbiome reshaping by antibiotics (MRA), FMT, and temporal and technical variability of human gut metagenomes (TTV) (see Methods and Supplementary Table 1). For each sample, we extracted *k*-mer features, calculated the pairwise Jaccard similarity scores of this sample against all other samples, except for those from the same individual, and generated a similarity distribution for which we fitted four models, including normal, truncated normal on interval [0,1], gamma, and beta distributions. Under the Kolmogorov–Smirnov (KS) test, we found that the beta distribution performed the best among the four distributions. (Supplementary Figure 1).

**Fig. 2:**
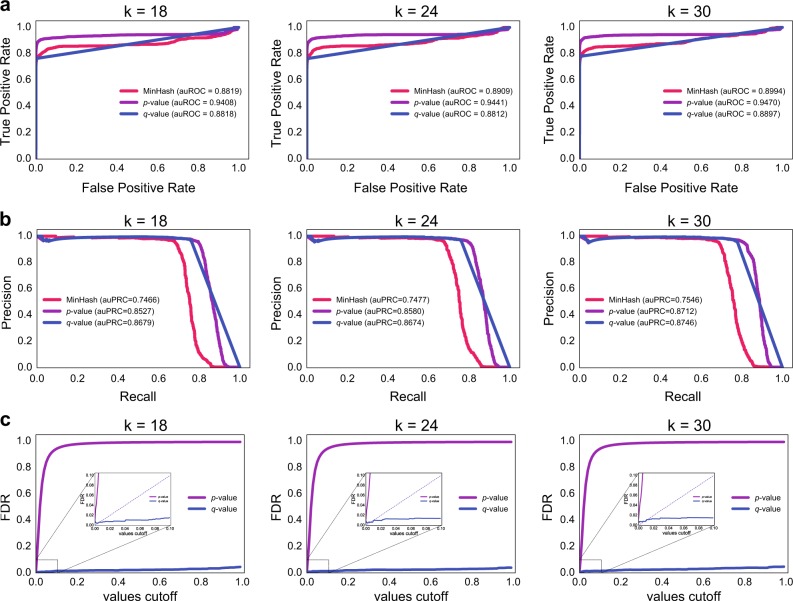
Overview the accuracy of GePMI using different values of *k*. **a** ROC curves of the three evaluation criteria: MinHash similarity, GePMI *p* value, and GePMI *q* value. **b** Precision-recall curves of the three criteria. **c** False discovery rate of using GePMI *p* values and *q* values FDR-corrected for PMI. MinHash was used with 10,000 hashes in all cases and 1 billion of sequenced bases were used per sample

To determine whether two metagenomic samples come from the same individual, we compared three metrics: (1) MinHash similarity, (2) GePMI *p* value, and (3) GePMI *q* value. From the ROC curve (Fig. 2a), we observed that GePMI *p* value outperforms the other two for any length of *k*, with the improved ROC values approximately between 0.05 and 0.06. Since the number of samples from the same individual was far less than the aggregate number of samples from different individuals, we also plotted the precision-recall curve. As shown in Fig. 2b, both GePMI *p* value and GePMI *q* value outperform the MinHash similarity by 0.11 and 0.12 on average. Since a query sample is tested against multiple samples in GePMI, we need to account for multiple testing and control the FDR. Here we used Benjamini and Yekutieli’s method (BY)^50^ for correction of *p* values and observed that FDR obtained by BY method was well controlled (Fig. 2c). The results of ROC, PRC, and FDR suggest that GePMI *q* value had the overall best performance, and thus we used the GePMI *q* values in the subsequent analysis.

There are three parameters to be considered in PMI: (a) the sequencing depth, denoted as *s*, the number of sequenced bases per sample, (b) the length of *k*-mer, denoted as *k*, (c) the size of hash table used for MinHash, denoted as *n*.^39^ We compared the GePMI *q* values for different values of *k* (15, 18, 21, 24, 27, and 30 because there are few common *k*-mers within genera when *k* ≥ 15^51^), *s* (10, 100, and 1000 millions), and *n* (1000 and 10,000) on PMI. It should be noted that we down-sample the bases of a sample from original “All” bases into 1000, 100, and 10 million bases. As shown in Table 1, the ROC value tends to improve with the increase of *s* except for the case of *k* = 15, and when *K* ≥ 18, the increase of *k* has little effect on the results. Another observation is that when *s* equals 1 billion, *n* = 1000 and 10,000 give similar results. We could have increased *n* = 100,000, but the cost of computational time and space is too big to be practical. If we increase *s*, many samples would not have enough bases to be included in the study. Considering the above observations, we set *s* = 1 billion, *k* = 18, and *n* = 10,000 as default parameters.

**Table 1:** The impact of three parameters on performance based on FDR-corrected *q* values generated by GePMI

*s***/***k* (of bases)	15-mer	18-mer	21-mer	24-mer	27-mer	30-mer
	Area under ROC curve (*n* = 1000/10,000 hashes)					
10 millions	0.7049/0.7741	0.6661/0.7370	0.6610/0.7486	0.6219/0.7446	0.6008/0.7508	0.6085/0.7553
100 millions	0.8146/0.8383	0.8274/0.8486	0.8150/0.8477	0.8064/0.8442	0.8176/0.8448	0.8067/0.8616
1 billion	0.7394/0.7417	0.8762/**0.8818**	0.8640/0.8818	0.8664/**0.8812**	0.8792/0.8871	0.8915/**0.8897**
All	0.6663/0.6773	0.8524/0.8625	0.8488/0.8623	0.8455/0.8665	0.8728/0.8714	0.8577/0.8762
	Area under precision-recall curve (*n* = 1000/10,000 hashes)					
10 millions	0.3657/0.4324	0.3731/0.4074	0.3609/0.4212	0.3358/0.4192	0.4094/0.4262	0.3639/0.4337
100 millions	0.7875/0.8052	0.8049/0.8243	0.7977/0.8230	0.7923/0.8202	0.7990/0.8203	0.7872/0.8322
1 billion	0.7348/0.7378	0.8637/**0.8679**	0.8528/0.8680	0.8518/**0.8674**	0.8297/0.8726	0.8755/**0.8746**
All	0.6505/0.6725	0.8014/0.8294	0.7854/0.7949	0.7779/0.7574	0.7117/0.7559	0.7468/0.7412

The two values per cell are results for *n* = 1000 /10,000 hashes in MinHash. Bold markers correspond to the results used in Fig. 2. The ‘All’ rows show results with all available data for each sample

### PMI before and after antibiotic treatment

**Fig. 3:**
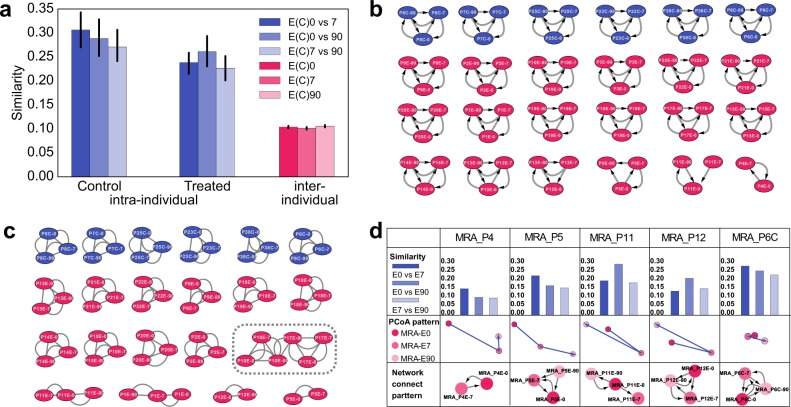
Similarities between metagenomic samples before and after antibiotic treatment. **a** The indigo bars represent similarity scores of two samples from the same individual (intra-individual) without antibiotic treatment (control) and with antibiotic treatment (treated), and the pink bars represent similarity scores of samples from different individuals (inter-individual). ‘E(C)0’, ‘E(C)7’, and ‘E(C)90’ represent samples before treatment, 7 days after treatment, and 90 days after the end of the treatment, respectively. **b** Local clusters of the network of MRA (microbiome reshaping by antibiotics) samples. A directed edge points from sample *a* to sample *b* if the *q* value of testing sample *a* against *b* is <0.001. **c** Similarity network of MRA samples. An edge connects two samples if the MinHash similarity of two samples is >0.199. The gray dashed box shows false positive pairs. **d** Some representative samples with MinHash similarity values, PCoA patterns (Supplementary Figure 3), and intra-individual sample networks. MRA_P4, MRA_P5, MRA_P11, and MRA_P12 are four subjects for whom the three samples are not fully connected, as shown in Fig. 3b, and MRA_P6 represents 1 of the 14 antibiotic-treated subjects (and all 6 control subjects) for whom all 3 samples are fully connected, also as shown in Fig. 3b

We next investigated whether individuals could be identified after medical treatments that are known to alter microbiomes. To accomplish this, we analyzed the MRA dataset consisting of 18 individuals taking drug Cefprozil (a cephalosporin antibiotic) and 6 controls.^45^ Each subject in this dataset had three sampling visits, right before treatment (E0), 7 days later (end of treatment, E7), and 90 days after the treatment (E90). Data from the same three time points (without treatment) was collected for the controls. In Fig. 3a, we plot the average pairwise similarity scores (intra-individual) among sampling visits from the same individual for the control and case groups, respectively, and those (inter-individual) among sampling visits from different individuals without distinguishing control and treated samples. Previous studies using resistant gene-related *k*-mers as features showed that the Jaccard intra-individual similarities in the control group were higher than those in the antibiotic treatment. In contrast, our results showed that sample pairs of the same individual, irrespective of the treatment, were much more similar to each other than the inter-individual pairs, indicating the effectiveness of the GePMI metric for PMI. In the inter-individual group, unrelated samples had consistently low similarity scores, irrespective of medical treatment.

We then pooled all five datasets together (612 samples in total) and applied GePMI to query each sample against all other samples for identification. Setting the *q* value cutoff to 0.001, we constructed a pairwise similarity network (Supplementary Figure 1). It should be noted that the edges in the network are directed because testing sample *a* against sample *b* may yield different result from testing sample *b* against sample *a*. Figure 3b shows the sub-network of the MRA dataset for an individual with three sampling visits; we can propose an ideal situation in which testing any sample against the other two in the same individual (intra-individual) shows significance, whereas testing any sample against the other two from other individuals (inter-individual) shows no significance. Since each individual has three samples, there will be six directed edges for each subject. 14 out of the 18 subjects from the antibiotic-treated group were correctly connected with *q* values <0.001, except for four subjects, MRA_P4, MRA_P5, MRA_P11, and MRA_P12 (Fig. 3b), where some sample connections were not detected. In total, we were able to predict 98 connections out of 108 within the antibiotic-treated group, achieving 90.74% accuracy, with no false positives. In comparison, the network constructed by the MinHash similarity scores with an optimal cutoff threshold (0.199) gave 2 false positives and missed 18 connections (83.3% accuracy) compared to GePMI (Fig. 3c).

Although it is well known that antibiotics can disrupt gut microbial communities,^52,53^ GePMI’s results indicate that most samples could still be correctly assigned to the original subjects after antibiotic treatment with no false positives. We noticed that some treated samples were no longer similar to the original samples. For example, for subject MRA_P4 (Fig. 3d), sample MRA_P4E90 was not significantly similar to either MRA_P4E0 or MRA_P4E7, showing that the antibiotic treatment, or other potential perturbations, had a significant impact on the subject, most likely transforming the microbiota into another state. In the study by Raymond et al.,^45^ samples MRA_P4E0 and MRA_P4E7 were both clustered into a subgroup that was dominated by *Prevotellaceae*, while MRA_P4E90 belonged to another subgroup with low diversity of *Bacteroidaceae*.^45^ Such changes could also be observed in subjects MRA_P5, MRA_P11, and MRA_P12. Overall, the results show that GePMI can robustly perform PMI.

### PMI before and after FMT

FMT is an operation that can restore healthy microbiota in patients. In this study, we obtained an FMT dataset containing samples from five patients (metabolic syndrome) transplanted with healthy donors’ microbiota (allogenic FMT).^47,48^ Samples were taken prior to transplantation (Day-0) and at multiple time points (Day-2, Day-14, Day-42, Day-84) afterwards. Three of the patients recovered (FAT_006, FAT_008, and FAT_020) and two retrogressed to disease (FAT_012 and FAT_015). Three of the five patients, FAT_006, FAT_008, and FAT_015, were transplanted with the same donor’s microbiota, while the other two were transplanted with different donors’ microbiota. Another five control patients, FAT_010, FAT_014, FAT_017, FAT_023, and FAT_024, were transplanted with their own microbiota (placebo-treated), and all of them remained unhealthy after FMT. For this dataset, we asked (1) whether the microbiome of an allogenic FMT recipient after treatment could be identified as that of the original, (2) whether samples of an allogenic-treated patient could be assigned to the donor, and (3) whether recipients transplanted with the same microbiota of the same donor could be matched.

For PMI, we counted a total of 206 ($10^\ast A_5^2 + A_3^2$) intra-individual-directed comparisons between samples, 200 from 10 subjects, each with 5 time-series samples, and 6 from comparing the three samples from the same donor. We set *q* value <0.001, and we found 128 related pairs and 5 donor pairs, including 86 out of the 100 pairs (86%) in the placebo-treated group but only 42 out of the 100 pairs (42%) in allogenic FMT group. The difference shows that the FMT had a noticeable impact on the microbiome.

**Fig. 4:**
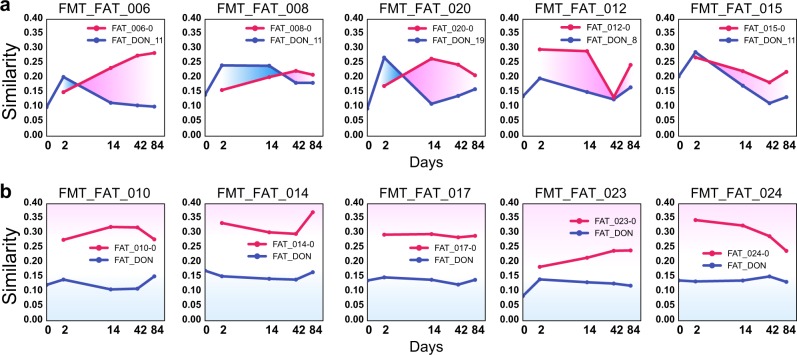
Dynamics of temporal changes of fecal microbiota transplant (FMT) sample similarity (using MinHash). **a** Change of sample similarity over time for the allogenic treatment group where five patients were transplanted with donor microbiota. The pink lines represent the similarities of transplanted samples with the sample before FMT. The indigo lines represent the similarities of transplanted samples with the donor’s sample. **b** Change of sample similarity over time for the autologous treatment group where five subjects were transplanted with their own microbiota. The pink lines represent similarities to the first sample, while the indigo lines represent the average similarity the samples of five separate donors, using FAT_DON as an average

Compared with the placebo-treated group, microbiomes of the allogenic FMT recipients were most similar to those of the corresponding donors, which posed a great challenge to individual identification.^48^ At Day-2 after FMT, four out of the five recipient samples were more similar to those of the corresponding donors than to their own baseline samples before FMT (Fig. 4a). However, by Day-84, all five recipient samples were more similar to their own baseline samples than to their corresponding donors. We observed that the samples from both FAT_012 and FAT_015, representing the retrogressed patients, at Day-2, right after transplant, had higher similarities to their original samples than those from the three recovered patients.

According to GePMI, on day 2, the first visit after FMT, the baseline samples from both FAT_012 and FAT_015 matched those of their corresponding recipients (*q* value < 0.001, Table 2). However, these two samples also matched the donor’s samples. In other words, the posttransplanted samples at day 2 were mixtures of two microbiomes; as such, these samples were similar to both the original and donor samples. Analysis of species-level OTUs also showed that FAT_012 and FAT_015 shared more species proportionally with those of the donors at day 2 when compared to the other three FMT subjects. In contrast, on the first visit after FMT, the microbiomes of the three recovered were not identified by GePMI to be significantly similar, either to their own or their donors’ microbiome; however, at later visits, their samples did match the baseline samples but did not match those of the donors, which can be explained as donor-specific strains that gradually disappeared in the recipients over time.^48^ As expected, samples of the placebo-treated subjects were not similar to those of the donors (Fig. 4b).

**Table 2:** GePMI similarity *q* values of FMT samples to self-baseline sample and to donor sample

GePMI (*q* value)	d2–d0	d14–d0	d42–d0	d84–d0	d0–dn	d2–dn	d14–dn	d42–dn	d84–dn
FAT_006	0.5119	0.0	0.0	0.0	1.0	1.0	1.0	1.0	1.0
FAT_008	1.0	0.0510	0.0064	0.0372	1.0	0.1051	0.1156	1.0	1.0
FAT_020	0.0031	4.752e-13	1.584e-13	1.583e-8	1.0	0.0362	1.0	1.0	1.0
FAT_012	1.517e-4	3.450e-4	1.0	0.0613	1.0	7.509e-4	1.0	1.0	0.4693
FAT_015	2.039e-4	0.0844	1.0	0.1182	1.0	9.532e-4	1.0	1.0	1.0
FAT_010	0.0	0.0	0.0	0.0	n/a	n/a	n/a	n/a	n/a
FAT_014	9.754e-7	8.483e-5	2.563e-4	3.275e-9	n/a	n/a	n/a	n/a	n/a
FAT_017	0.0	0.0	0.0	0.0	n/a	n/a	n/a	n/a	n/a
FAT_023	0.0	0.0	0.0	0.0	n/a	n/a	n/a	n/a	n/a
FAT_024	2.113e-5	1.558e-4	0.0068	0.1560	n/a	n/a	n/a	n/a	n/a

Last five subject did not transplant any donor’s microbiota

*d2–d0* day 2 to baseline for the same individual, *d2–dn* individual to the donor, *n/a* not applicable

In summary, transplantation with other person’s microbiota does, indeed, affect the recipient’s microbiome. To our surprise, the results of data analysis using GePMI show (1) that samples from failed transplantation initially matched samples of both recipients and donors but deviated from both at the end, and (2) that samples from successful transplantations did not initially match those of either recipients or donors, but similarity to the recipient’s original samples did return at the end of the experiment.

### Robustness of PMI: technical variability and complex treatment

**Fig. 5:**
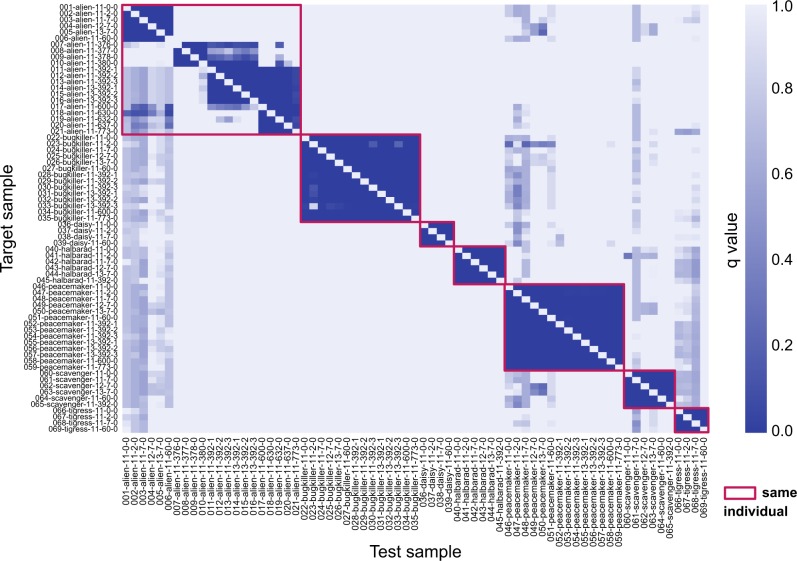
The heat map of metagenomic samples from seven individuals in the TTV dataset^21^ and the asymmetric *q* values between test samples and target samples. The sample label's convention is sample number-individual’s nickname-DNA sampling method number-day-repeat

The TTV dataset consists of 69 metagenomic samples of 7 individuals (Alien, Bugkiller, Daisy, Halbarad, Peacemaker, Scavenger, Tigress) from Voigt et al.^21^ who investigated the impact of temporal sampling and DNA storage methods on metagenomic sequencing. Time series and replication data were produced for each person. Analysis of this dataset by GePMI showed that DNA sampling and storage methods had very little influence on PMI, as all samples from the same individual at the same time matched. As an example, alien-11, alien-12, and alien-13 all came from the same individual at the same sampling time, but different sampling methods were carried out. Nevertheless, their pairwise similarities were high and GePMI test statistics were significant (Fig. 5).

However, temporal variation does have some impact on PMI. As shown in Fig. 5, excluding samples from subject Alien, GePMI identified 410 out of the 480 sample pairs (91.52%) using *q* value <0.001, but for Alien, only 119 out of the 420 sample pairs could be identified. The metagenomic samples of Alien not only reflected technical variability, using different DNA extraction methods, but also contained samples with antibiotic treatment (Ceftriaxone, a broad-spectrum antibiotic) and bowel cleansing. We had previously shown in the MRA dataset that antibiotic treatment did not change PMI for most individuals, but alien’s post-antibiotic treatment samples could not be matched to pre-antibiotics samples because the adjusted GePMI *q* values were all >0.001 (Fig. 5 and Supplementary Figure 4). However, the samples after bowel cleansing (days 600–773) could be matched to those before bowel cleansing but not after antibiotic treatment, a finding that was not discovered in the original study. This suggests that GePMI is more accurate than the standard distance/similarity metrics.

It should be noted that GePMI produces two asymmetric values for each pair of samples. Sample *a* may be statistically similar to sample *b*, but the reverse may not be true. In this dataset, the Alien sample at day 600 was statistically similar to the sample at day 392, but the sample at day 392 was not statistically similar to the sample at day 600 because the same similarity value was tested against two different distributions, thus producing two different results (Supplementary Figure 4). The samples collected at day 392 were most likely still in the recovery/transition status after antibiotic treatment, while samples at day 600 had regained their stability.^21^ Moreover, since samples at the first 60 days were not statistically similar to the samples at later days, we conclude that the treatment did change the genome composition of the microbial community.

### Some extensions of GePMI applied to individual identification

Besides the TTV dataset, the samples listed are from different DNA processing methods. A few of the metagenomic sequencing data that we collected were sequenced by multiple sequencing platforms, with different read length and using different DNA extraction methods. For example, two subjects, s159490532 and s159591683 from the HMP dataset, were sequenced by both 454 GS FLX Titanium and Illumina Genome Analyzer II. The read length and error models were different for these two sequencing platforms. However, both resulted in *p* values close to zero when testing 454 samples of s159490532 against the inter-individual distribution of Illumina’s samples and the same was true for the reverse test. Therefore, we can confidently say that they were from the same subject. We obtained a similar result for subject s159591683. Although it was a small test, it suggests that sequencing platforms did not affect the accuracy of the identification if the error rates were small. As we have shown in the TTV dataset, DNA extraction methods had little effect on PMI.

For comparison with read-based similarity measures, we mapped the all 612 down-sampled reads of 5 collected datasets to the NCBI microbes non-redundant database using DIAMOND blastx,^54^ computed the species abundance, and calculated similarities based on Bray–Curtis metric.^55^ The accuracy of species-based similarity is 0.8308 (auROC), worse than that of the *k*-mer-based GePMI method (0.8818). However, the difference measured by auPRC was much larger (0.3759 vs. 0.8679). Hence, using species as features for PMI appears to have a larger false positive rate. Meanwhile, we also assembled these reads by MEGAHIT^56^ and computed each sample’s contig similarity using GePMI with 18-mers and 10,000 hashes. The accuracy, shown in supplementary Figure 5a, is slightly lower with auPRC = 0.7513. Compared to applying GePMI on the raw reads directly (Fig. 2), assembling the reads first does not help GePMI, since a lot of sequence variations are lost during assembly.

We also tested our approach on a 16S rRNA dataset. In general, different projects may sequence different 16S rRNA gene hypervariable regions, making it difficult to compare across different projects. Nevertheless, we obtained the HMP gut dataset,^26^ which consists of 325 samples for 222 individuals, 121 with one visit, 99 with two, and 2 with three. We used a 97% similarity threshold for OTU definition (11,752 OTUs in total). To identify samples from the same individuals, we applied both Bray–Curtis similarity metric (auROC = 0.9401, auPRC = 0.4715) and GePMI (auROC = 0.9604, auPRC = 0.5282) on this dataset, and the results showed that GePMI performed slightly better. However, the precision value for a fixed recall rate was worse than that using metagenome sequencing data. This is because 16S rRNA gene sequences contain much less information than genome sequences. However, considering that 16S rRNA sequencing costs less, it could serve as a cheaper alternative for some special application scenarios.

We also tested GePMI on data collected from tongue dorsum in the HMP project.^26^ The dataset consists of 48 subjects with only one sample visit, 38 subjects with two, and 4 subjects with three, for a total of 136 samples. Applying GePMI to this dataset using the same parameters as those in the gut dataset (see Supplementary figure 5b), we obtained auROC score 0.8749 and auPRC score 0.8584. Using threshold *q* value ≤0.1, 52% of the samples could be correctly identified with 2% false positives. The results were not as good as those for the gut datasets. A possible explanation is that microbiome of tongue dorsum has much bigger variation than that of the guts. However, oral samples are easy to obtain and can be used to explain some oral diseases, which provide potential possibilities for individual identification through oral metagenomic samples.

## Discussion

In this paper, we demonstrated that a personal microbiome could be uniquely identified with high accuracy across several different metagenomic data sets. We used Jaccard similarity, implemented with MinHash approximation,^39^ to measure pairwise similarity. In GePMI, Jaccard similarity was tested against the target sample’s inter-individual distribution under the null hypothesis that pairwise similarity arises from the target sample’s inter-individual similarity distribution. The final score is an adjusted *q* value for PMI. We proved that most metagenomic samples can be identified, even after clinical treatments, such as antibiotic treatment and FMT, although we saw some cases where the microbiome had moved to a new state no longer similar to the original one. In summary, the human microbiome has obvious personal characteristics, and individual samples can be uniquely identified in most cases.

For the MinHash strategy, length of *k*-mers and the number of minimum hash values can influence similarity calculations. In general, longer *k*-mers would be more sensitive to strain variations, but they could also be more affected by sequencing errors. On the other hand, the number of features increases exponentially with *k*, meaning that we would need to store a larger hash table. Before the similarity calculation, we do subsampling to ensure that the samples have the same number of *k*-mers. However, without the microbial genome references, it is hard to identify strains underlying each microbiome.^57^ In general, our results indicate that *k*-mers of size ≥18 were specific enough to characterize a microbiome.

Temporal and microbiome variability can interfere with individual identification. Our study showed that temporal variation had a major impact on the consistency of our identification. As an extreme example, human gut microbiota during infancy is completely different from that of older age^58^; therefore, it is unlikely an adult’s gut microbiome can be matched to his/her infant gut microbiome. Inter-individual microbiome variation may come many sources.^20,59^ We demonstrated that clinical treatments could change microbial communities such that some individuals’ samples might become, in effect, a new “subject” similar to neither self nor to others.

We compared GePMI to Metagenomic Codes that used sequence markers as the features for 50 individuals with two visiting stool samples and obtained a true positive rate (individuals who were identified) of 86%, that is, 43 individuals could be uniquely identified by 6–8 marker-based codes.^30^ For GePMI, each sample was tested against the other 99 samples. Following the same definition of Metagenomic Codes judgment rules, GePMI with *q* value ≤0.1 produced 97% true positive rate with no false positive (Supplementary figure 5c), better than that using the Metagenomic Code. While the idea behind the Metagenomic Code is quite elegant, the proposed “body site-specific metagenomic codes” could be very hard to define as more samples are added into the pool. On the other hand, because human intestinal microbiome may experience significant changes due to growth, antibiotic treatment, and FMT, defining metagenomic codes could be very challenging.

Whether PMI is based on the uniquely recognizable “fingerprints”^30^ or based on the fact that samples from intra-individual are significantly more similar than those from inter-individuals, we believe that the personalized microbiome era has already arrived. In the past decades, scientists have focused on defining the core microbiota for each environment^18,60–62^; however, the focus has been shifted to the study of specific features of each individual’s microbiota and their relationship with the environment^20,22,46,63,64^ and the study of evolution of microbial communities over time through time-series samples.^23,33,65,66^ A healthy individual’s microbial community is robust against external perturbations, demonstrated by its ability to maintain homeostasis and to recover from disease.^52,58,67^ However, the exact process and path of its moving between a healthy state and a disease state remains a mystery, although we can distinguish the microbiota in an individual’s health state from that in the disease state. PMI may be applied to such cases to help us to understand this process and to design efficient intervention methods to cure the disease. If we observe that an individual’s two metagenomic samples can no longer be linked through our method, this indicates that the microbial community has changed its state. Although we cannot determine whether or not the changes may lead to disease, GePMI can serve as a monitoring tool (independent of obvious clinical manifestations) for further examination of potential diseases. Overall, GePMI provides a computational framework to measure personalized microbiota, and we believe that it can be applied to a wider range of applications in precision medicine.

## Methods

### Data

DNA sequencing reads from 634 human fecal samples were downloaded from the HMP,^26^ MetaHIT,^16^ MRA,^45^ FMT,^47^ and TTV.^21^ After quality control by FaQC^68^ to remove low quality reads, we mapped reads to hg19 genome reference ^69^ to remove human genome reads. Samples with at least 1 billion bases were selected, and as a result, 612 samples were retained for further analysis, including 248 samples from 138 individuals in HMP, 168 samples from 119 individuals in MetaHIT, 72 samples from 24 individuals in MRA, 55 samples from 13 individuals in FMT, and 69 samples from 7 individuals in TTV. Details can be found in Supplementary Table 1.

### GePMI: personal microbiome identification

MinHash is a locality-sensitive hashing method for rapid calculation of similarity between two sets based on Jaccard similarity. In this paper, we used khmer^70^ for removing low abundance *k*-mers and we computed approximated Jaccard similarity for every pair of samples by using sourmash^39^; they were run as:

- *k*-mers error trimming: trim-low-abund.py –k 18 –C 2 sample.fa > subject-sample.fa
- creating signatures: sourmash compute -k 18 -n 10000 subject-sample.fa -o subject-sample.sig
- building distance matrix: sourmash compare -k 18 --csv output.csv -o output *.sig

Similarity values between a sample and those of all other individuals form an inter-individual similarity distribution for this specified sample. We set the null hypothesis that the similarity value of a test sample to a target sample is drawn from the target sample’s inter-individual similarity distribution, i.e., the test and target samples came from different subjects. The rejection of the null hypothesis means that the test and the target samples are from the same subject.

The similarity values range from zero to one. We checked which of the following distributions best fit the data: (1) a truncated normal distribution, (2) a gamma distribution (because the Jaccard similarity for low diversity samples is usually near zero), and (3) a beta distribution. We performed KS test for these three distributions. Let *X* be the set of inter-individual similarities. Under one sample’s distribution, the *p* value that the test sample similarity to the target sample is equal to

$$p = {\mathrm{Pr}}(X \ge s|H_0)$$

where *H*_0_ is the null hypothesis. By using the *p* value, we can determine whether two samples are from the same subject or not. To control the FDR in multiple testing, Benjamini and Yekutieli’s method was used to transform *p* values to *q* values.^50^ By combining both the *p* and *q* values, we can determine the subject to which a test sample belongs in the database. GePMI script can be run as:

- python GePMI.py -i output.csv -p 0.001 -q 0.01 -s 0 -o outputDir –t
-p, -q, -s are the parameters (*p* values, *q* values, and similarity) to set thresholds for hypothesis testing in the final results.

### Availability statement

Method availability: https://github.com/princello/GePMI.

## Electronic supplementary material

Supplementary Table 1

Supplementary Figure Legends

## Data Availability

The datasets analyzed during the current study are available in ENA repository (BioProject accession nos. PRJEB12357, PRJEB8094, PRJEB2054, PRJEB1220, and PRJEB8347) and HMP dataset (https://www.hmpdacc.org/HMIWGS/all/).

## Electronic supplementary material

**Supplementary information** accompanies the paper on the *npj Biofilms and Microbiomes* website (10.1038/s41522-018-0065-2).
