# Author Correction: GePMI: a statistical model for personal intestinal microbiome identification

**DOI:** 10.1038/s41522-018-0071-4

**Published:** 2018-11-07

**Authors:** Zicheng Wang, Huazhe Lou, Ying Wang, Ron Shamir, Rui Jiang, Ting Chen

**Affiliations:** 1 MOE Key Laboratory of Bioinformatics and Bioinformatics Division, BNLIST and Department of Automation, Tsinghua University, 100084 Beijing, China; 2 Bioinformatics Division, BNLIST and Department of Computer Science and Technology, Tsinghua University, 100084 Beijing, China; 3 Department of Automation, Xiamen University, 361005 Fujian, China; 4 Blavatnik School of Computer Science, Tel-Aviv University, Tel Aviv, Israel

**Correction to**: *npj Biofilms and Microbiomes* 10.1038/s41522-018-0065-2, Published online 04 September 2018

In the original version of this Article some funding information was missing from the Acknowledgements. This has now been corrected in the PDF and HTML versions of the Article.

