# Supplementary material for: GePMI: A statistical model for personal intestinal microbiome identification
Source: NPJ Biofilms Microbiomes. 2018 Sep 4;4:20. doi: 10.1038/s41522-018-0065-2 (PMC6123480; doi:10.1038/s41522-018-0065-2)
Supplement: Supplementary file 2 — Supplementary Figure Legends [file 41522_2018_65_MOESM2_ESM.docx]

__

Supplementary Figure 1. (a) Kolmogorov-Smirnov test for four methods to fit each sample’s inter-individual similarity distribution. The average *p*-values are 0.296±0.285, 0.310±0.290, 0.293±0.297, and 0.656±0.290 for normal, truncated normal, gamma and beta distributions, respectively. (b) The average statistics of Kolmogorov-Smirnov test are 0.0464±0.0180, 0.0457±0.0178, 0.0530±0.0529, and 0.0301±0.0106 for normal, truncated normal, gamma and beta distributions, respectively. Boxes in boxplots show 25%-75% percentile and whiskers show 5%-95% percentile.


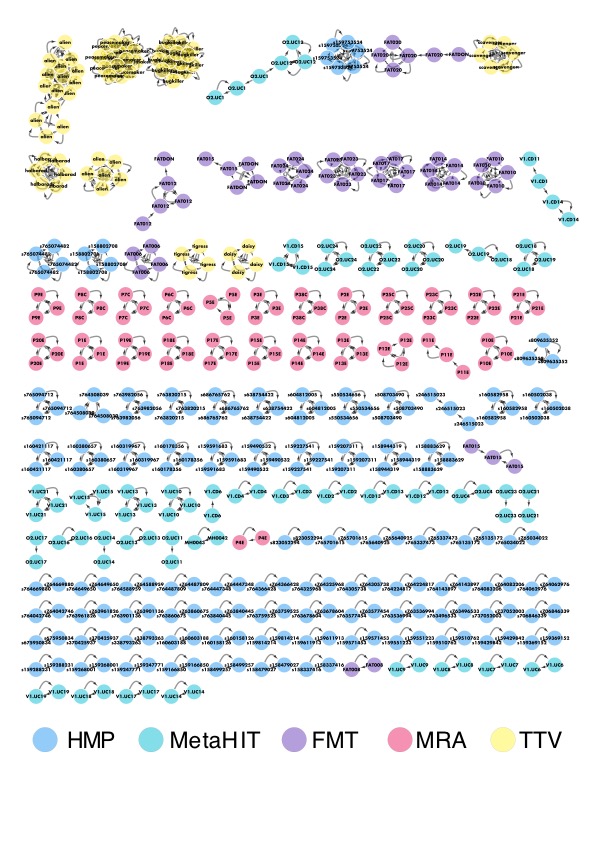


Supplementary Figure 2. Network of all samples. An edge from sample *a* to be indicate that the *q*-value of testing if *b* is similar to a is <0.001. HMP, MetaHIT, FMT, MRA, TTV are five collected datasets.


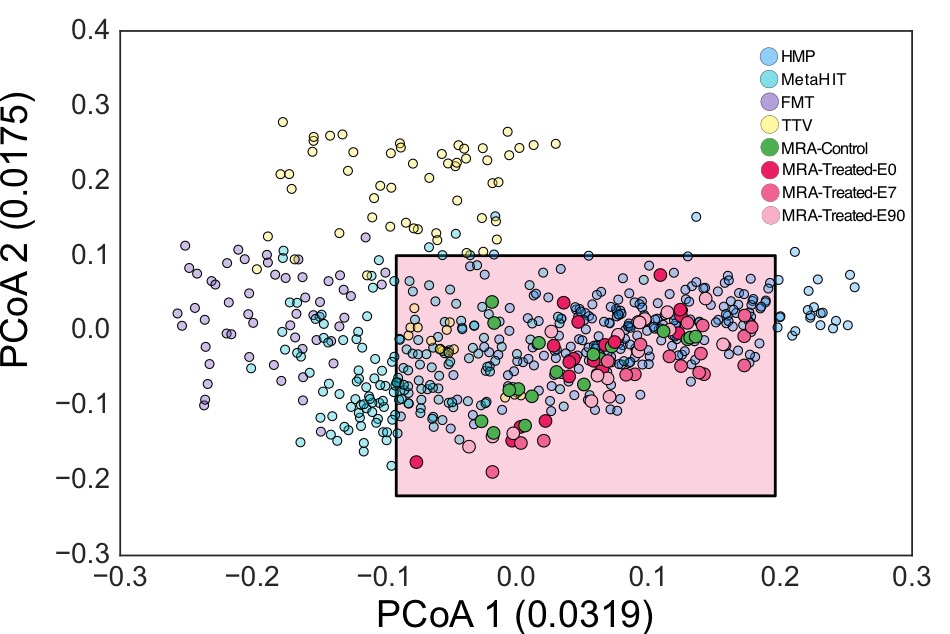


Supplementary Figure 3. PCoA plot using pairwise similarities of all collected samples, MRA stands for antibiotics treatment data.

Supplementary Figure 4. Heat map of metagenomic samples from 7 individuals in the TTV dataset^21^. MinHash similarities of the test samples to the target samples are symmetric and aggregate along the diagonal. alien, bugkiller, daisy, halbarad, peacemaker, scavenger and tigress are the names of subjects.

Supplementary Figure 5

The performance of GePMI on different data sets. a) 612 gut metagenomic samples after assembly. b) 136 tongue dorsum metagenomic samples from HMP project. c) 100 gut metagenomic samples from HMP 50 subjects^26^.

Supplementary Table 1

Detail of each sample
